# Is work engagement associated with healthier dietary patterns? A cross‐sectional study

**DOI:** 10.1002/1348-9585.12149

**Published:** 2020-07-25

**Authors:** Hoichi Amano, Yoshiharu Fukuda, Megu Y. Baden, Ichiro Kawachi

**Affiliations:** ^1^ Department of Social and Behavioral Sciences Harvard T.H. Chan School of Public Health Boston MA USA; ^2^ Graduate School of Public Health Teikyo University Tokyo Japan; ^3^ Department of Nutrition Harvard T. H. Chan School of Public Health Boston MA USA

**Keywords:** employee, healthier dietary behavior, Japan, work engagement

## Abstract

**Objectives:**

Work engagement is defined as a positive, fulfilling feeling about one's job and is associated with higher productivity and morale. We performed a cross‐sectional study to investigate whether work engagement is related to healthier dietary behaviors among Japanese workers.

**Methods:**

The present study was part of the Japanese Study of Health, Occupation, and Psychosocial Factors Related to Equity. A validated food frequency questionnaire was used to evaluate daily nutritional intake. The following seven nutrients were considered: salt intake, dietary fat (saturated fatty acids, monounsaturated fatty acids, and polyunsaturated fatty acids), dietary fiber, folate, and isoflavone. Multivariable linear regression analysis was performed, adjusting for job stress, psychological distress, and other confounders. The baseline survey inquired about work engagement among 2,233 employees of 12 workplaces in Japan, representing a range of industries.

**Results:**

The mean age of the sample was 43.4 ± 9.7 years and 89.7% of the participants were male. The mean score of work engagement was 2.9 ± 1.0. Higher work engagement was significantly positively associated with higher salt intake (β = 0.17, *SE* = 0.06, *P* = .006), monounsaturated fatty acids (β = 0.29, *SE* = 0.13, *P* = .03), polyunsaturated fatty acids (β = 0.28, SE = 0.09, *P* = .001), dietary fiber (β = 0.23, SE = 0.09, *P* = .012), and folate (β = 10.2, *SE* = 2.9, *P* = .005) consumption, but not saturated fatty acid (β = 0.16, *SE* = 0.11, *P* = .13) or isoflavone (β = 0.64, *SE* = 0.36, *P* = .072).

**Conclusion:**

The present study suggested that higher work engagement is associated with a healthier pattern of dietary behaviors among workers. Improving work engagement may be a novel target for workplace health promotion.

## 1 INTRODUCTION

Work engagement is defined as “a positive, fulfilling state of mind” in relation to one's work, which is described by “vigor, dedication, and absorption.”^1^ A close association has been reported between work engagement (and its opposite concept, burnout) and job performance.^2^ The Job Demands‐Resource model (JD‐R model) provides a theoretical platform to understand these associations.^2^ According to the JD‐R model, while burnout is linked to job demands (ie workload and time pressure), work engagement is influenced by the available resources on the job (ie decision latitude, supervisor support, co‐worker support, and extrinsic rewards). Previous studies primarily focused on burnout as a predictor of health impairment, while work engagement has been mostly looked at as a predictor of job performance and employee turnover.^2^, ^3^ During the past decade, however, research on work engagement has shifted focus from job performance and employee turnover to health‐related outcomes among employees.^3^, ^4^ Studies to date have revealed that work engagement is related to lower risk of ill‐health, assessed using the Brief Job Stress Questionnaire,^3^ as well as lower levels of inflammation, assessed with high‐sensitivity C‐reactive protein.^5^ However, despite the accumulating evidence in support of work engagement associations with health, the association between work engagement and health behaviors remains under‐examined.^6^ To the best of our knowledge, there has been only one previous study that investigated the association between work engagement and dietary behaviors.^6^ While this study focused only on fish consumption, we investigated a more comprehensive profile of nutritional intakes.

Previous studies investigating an association between positive psychological well‐being and healthy diet behaviors provide some clues.^7^, ^8^ That is, people with higher positive psychological well‐being are more likely to have healthy dietary behaviors. Work engagement can be conceptualized as an aspect of positive psychological well‐being. Work engagement is also linked to life satisfaction or “happiness,”^3^ and can be considered a reflection of positive psychological well‐being in the occupational field. The two theoretical reasons why positive well‐being is associated with heath behaviors is because of: (a) the absence of negative psychological states (eg depressed affect), which have been consistently linked with an unhealthy pattern of behaviors—for example, depressed individuals often “self‐medicate” with nicotine or alcohol as a means of coping with their emotional state. They may also engage in patterns of “comfort eating,” that is, consumption of energy‐dense foods high in fat and sugar. Of course, work engagement can be associated with less negative psychological states; and (b) a positive orientation toward work enhances the individual's motivation to invest in their long‐term health. A previous study suggested that work engagement was linked to higher life satisfaction.^3^ Simply stated, an individual who is positively engaged in his/her work has increased reason to stay healthy in order to continue doing what he/she enjoys in life. Our hypothesis is that life satisfaction increases the individual's motivation to invest in health‐promoting behaviors in order to prolong their enjoyment of life and happiness. Conversely, people with low life satisfaction often say that they have lost the motivation to live as long as possible. Hence, the causal pathway is from: work engagement → life satisfaction → investment in health. To bolster our reasoning, previous studies have shown that those with psychological well‐being tend to have healthy dietary behaviors. The association between positive states of mind and healthy nutritional habits is likely to be bi‐directional. On the one hand, a more positive state of mind strengthens a person's resolve to stay healthy and invest for the future—for example, by keeping fit and eating well. Conversely, negative states of mind are associated with maladaptive stress‐coping behaviors, such as excess drinking, smoking, and “comfort eating” (over‐consumption of high‐fat, high‐sugar, high‐salt foods).^9^ The reverse pathway—that is, healthy eating can promote positive states of mind—has also received attention in recent years.^10^ Again, work engagement is considered to a kind of positive states in the occupational field. There are reasonable grounds, therefore, to hypothesize that work engagement is associated with nutritional habits. In this study, we hypothesized that people with higher work engagement—associated with goal setting, self‐efficacy, motivation, and self‐regulation—are more likely to adopt healthier dietary behaviors.

Accumulating evidence supports that a healthy dietary pattern – for example, as assessed by the Alternate Healthy Eating Index, the Alternative Mediterranean Diet Score, and the Dietary Approaches to Stop Hypertension (DASH) diet score—is related to a lower risk of chronic diseases.^11^ Common food groups contributing the most to healthy dietary patterns include whole grains, vegetables, fruits, nuts, and fish. In turn, consumption of these food groups is associated with higher intake of vitamins, polyphenols, polyunsaturated fatty acids (PUFA), and dietary fiber, as well as lower intake of saturated fatty acids (SFA) and salt. Accordingly, we hypothesized that work engagement is correlated with higher consumption of dietary fiber, isoflavone, and PUFA, as well as lower intakes of SFA and salt.

The reasons why we choose these factors are as follows:

1. **Salt**

According to the World Health Organization (WHO), the fourth highest risk factor for non‐communicable disease mortality worldwide is high salt intake. Excess salt intake is associated with higher risks for hypertension, stroke, cardiovascular diseases, and end stage kidney disease.^12^, ^13^ WHO recommends a salt intake of 5 g or less per day for the general population.^14^

- **Lipids**

Higher SFA intake and lower monounsaturated fatty acids (MUFA) or PUFA intake is associated with CVD. A meta‐analysis showed that replacing SFA with PUFA or MUFA will lower cvd events.^15^

- **Dietary fiber**

Accumulating evidence shows that people with higher dietary fiber intake have lower risk of CVD^16^ and Type 2 diabetes.^17^ Although dietary fiber intake of 19‐20 g/d for men and 17‐18 g/d for women is recommended,^18^ the mean dietary fiber intake among Japanese people was 14.5 g/d in 2015.^19^ Dietary fiber is abundant in whole grains, fruits, and vegetables and increased intake of fiber is recommended because of its protective effect against CVD.

- **Folate**

Folate is an important regulator of homocysteine metabolism. Increased folate intake reduces serum homocysteine levels.^20^ Previous meta‐analyses demonstrated that folate intake was linked to a decreasing trend in stroke risk.^21^ Folate is also abundant in many fruits, vegetables, and legumes.

- **Isoflavone (polyphenol)**

Polyphenols can be divided into four or more subgroupings based on the number of phenol rings they contain and the structural elements that bind these rings to one another. Isoflavones belong in the flavonoids group. In this study, we focused on genistein (a kind of Isoflavones) as a marker of polyphenol intake. Isoflavones are rich in soy products such as tofu and Natto.

Polyphenols have a positive effect on fasting blood glucose, total cholesterol, and low‐density lipoprotein‐cholesterol.^22^ They are also considered to have anti‐inflammatory and antioxidant properties.^22^

## 2 METHODS

### 2.1 Data source

We analyzed cross‐sectional data collected in an occupational cohort study on social class and health in Japan (Japanese Study of Health, Occupation, and Psychosocial Factors Related Equity: J‐HOPE). The baseline survey was collected from April to June 2011.^5^ The study population consisted of employees working for 12 workplaces, representing a wide variety of industries: information technology, hospitals/medical facilities, manufacturing, pharmaceutical, service industries, transportation, and housing sales. These companies were selected to represent Japan's industrial structure, and the data used in J‐HOPE were collected from the companies that agreed to participate. Data were mainly collected during regular employee health check‐ups. In Japan it is legally mandated that employers provide annual health check‐ups for all employees where screening is offered for metabolic syndrome, cancer detection, etc Our questionnaire was administered to employees presenting for their annual check‐up. The original sample consisted of 10 742 responders in the first wave (response rate: 77%).

### 2.2 Assessment of work engagement

The nine‐item Japanese version of the Utrecht Work Engagement Scale (UWES‐9) ^23^, ^24^ was used to assess work engagement at baseline. The UWES‐9 includes three domains which examine vigor (three items), dedication (three items), and absorption (three items) on a seven‐point Likert response scale ranging from 0 (never) to 6 (always/every day). We used the average score of the nine items to assess work engagement. The UWES‐9 was translated into Japanese with acceptable internal consistency and reliability, as well as factor and construct validity.^24^ Cronbach's α coefficient for the total score was 0.94.

### 2.3 Dietary intake

Dietary habits during the preceding month were assessed with a validated, brief, self‐administered dietary food frequency questionnaire (BDHQ).^25^ The BDHQ is a four‐page structured questionnaire that asks about the consumption frequency of a total of 56 food and beverage items commonly consumed by the general Japanese population, with specified serving sizes described in terms of the natural portion or the standard weight and volume measurement of servings. The BDHQ was developed based on a more comprehensive (16‐page) version of a validated food frequency questionnaire.^26^

The validation of the BDHQ was performed by using 16‐day weighed dietary records as the gold standard, Pearson correlation coefficients for folate intake in 92 Japanese men and 92 Japanese women aged from 31 to 76 years were 0.50 and 0.62, respectively.^25^ Adjusted folate intake was calculated as daily folate intake divided by daily total energy intake (1000 kJ). Responses to the BDHQ were checked for completeness and, where necessary, clarified by direct questioning of the participant.

### 2.4 Demographic and socioeconomic variables

Age, sex, and highest educational attainment were self‐reported in the 2011 survey. We used educational attainment, occupation, and annual household income as indicators of socioeconomic statutes (SES). Education level was categorized into two groups: ≤12 years or >12 years of formal education. Occupations were categorized into four groups: Manager, Non‐Manager, Manual, and Others. Annual household income was considered as the sum of income from each family member, and the number of family members was assessed based on a self‐administered questionnaire. Each participant was asked to indicate to which of six income levels their household income belonged: (a) <3.0 million JPY/year, (b) 3.0–4.99 million JPY/year, (c) 5.0–7.99 million JPY/year, (d) 8.0–9.99 million JPY/year, (e) 10.0–15.0 million JPY/year, and (f) >15.0 million JPY/year. For the analysis, the average household income of each category was used and was equalized for household size by dividing by the square root of number of household members.^27^

### 2.5 Job stress and support

The short version of the Job Content Questionnaire (JCQ) was adapted and validated for the Japanese population by Kawakami et al^28^ The JCQ is a standardized instrument used to assess social and psychological characteristics of jobs based on the theoretical model developed by Karasek.^29^ It comprises 22 questions with response options ranging from “strongly disagree” to “strongly agree” scored on a Likert scale from 1‐4. The scale to measure social support comprised eight questions on relationships with co‐workers and supervisors. These variables were coded according to the Job Content Questionnaire User's Guide. Scores for each quartile on job demand variables were calculated; scores in the top quartile were labeled the high (reference) group. The bottom quartile was the low group, and scores in the middle quartiles were collapsed into the medium group. The job control and social support scores were similarly divided into three groups.^28^, ^29^, ^30^

### 2.6 Psychological distress

We used K6 to assess psychological distress. K6 is a six‐item self‐report questionnaire designed to screen for mood and anxiety disorders. A cut‐off point of five, which is what we used in this study, has been used in screening for clinical depression.^31^, ^32^

### 2.7 Outcomes and definition of healthy diet pattern

Accumulated evidence has investigated an association between healthy dietary pattern and health outcomes.^11^ Healthy dietary patterns tend to have more nutrients, vitamins, PUFA, and dietary fiber, and less SFA and salt.^12^, ^13^, ^14^, ^15^, ^16^, ^17^, ^18^, ^19^, ^20^, ^21^, ^22^ The following seven nutritional markers were included in our analysis: salt intake, dietary fats (SFA, (MUFA, and PUFA), dietary fiber, folate, and isoflavone.

### 2.8 Assessment of confounders

The followed factors were considered as potential confounders between work engagement and healthy behaviors; age, sex, household income, education, occupation, job stress, support of supervisor and colleagues, K6, workplace, and total energy intake.

According to the JD‐R model, the demands and level of control associated with the job, as well as the support of the supervisor and colleagues, are all associated with work engagement. We therefore controlled for these job characteristics as potential confounder.

In addition, previous studies showed that higher K6 scores are correlated with low work engagement.^6^ This may be because low work engagement leads to more psychological distress, or alternatively, workers experiencing high distress are less likely to be engaged. Since psychological distress is also robustly correlated with poor health behaviors, we considered K6 scores as a potential confounder of the relation between work engagement and health behaviors.^33^

### 2.9 Statistical analysis

A total of 2265 participants who had BDHQ data were enrolled in this study. We excluded participants who did not provide responses to the survey on work engagement, had a history of CVD or cancer at baseline, or had missing data (N = 32). The final analytic sample comprised 2233 participants.

Participant characteristics at baseline are summarized in Table 1. Continuous data were expressed as mean (standard deviation), and categorical data were expressed as percentages. To estimate daily nutritional intake, multiple regression analysis was used to calculate the standardized regression coefficients (β) and scandalized error (*SE*) by work engagement after adjusting for age, sex, household income, education, occupation, job stress, support of supervisor and colleague, K6, workplace, and total energy intake (Table 2).

**TABLE 1:** Participant characteristics

Age (years)	43.4 (9.7)
Sex (male, %)	89.7
Socioeconomic status	
Years of education	
12 or less (%)	32.4
Occupation	
Manager (%)	22.8
Non‐manual (%)	47.0
Manual (%)	21.2
Others (%)	9.0
Household income (million JPY/year)	444 (188)
Job stress and support	
Demand	32.1 (5.3)
Control	69.1 (9.5)
Supervisor support	11.4 (2.3)
Colleague support	11.4 (1.91)
Psychological stress	
K6	5.1 (4.6)
Work engagement	2.9 (1.0)
Nutrition	
Total daily Energy (kcal/d)	1843 (570)
Salt (g/d)	10.4 (3.2)
Lipid	
Saturated fatty acid (g/d)	12.7 (5.3)
Monounsaturated fatty acid (g/d)	18.0 (6.9)
Polyunsaturated fatty acid (g/d)	12.8 (5.3)
Dietary fiber (g/d)	10.1 (4.2)
Folate (μg/d)	295.8 (126.9)
Isoflavone (μg/d)	16.5 (13.1)

Note

Continuous data were expressed as mean (standard deviation), and categorical data were expressed as percentages.

**TABLE 2:** Standardized regression coefficients (β) and scandalized error (SE) to estimate daily nutrition by work engagement

	Salt (g/d)		Lipid (g/d)						Dietary fiber (g/d)		Folate (μg/d)		Isoflavone (μg/d)	
	β (*SE*)	*P*‐value	SFA		MUFA		PUFA		β (*SE*)	*P*‐value	β (*SE*)	*P*‐value	Genistein	
			β (*SE*)	*P*‐value	β (*SE*)	*P*‐value	β (*SE*)	*P*‐value					β (*SE*)	*P*‐value
Unadjusted Model														
Work engagement (per 1 score)	0.26 (0.07)	<.001	0.41 (0.11)	<.001	0.6 (0.15)	<.001	0.45 (0.1)	<.001	0.44 (0.09)	<.001	17.1 (2.82)	<.001	1.02 (0.29)	<.001
Adjusted Model														
Work engagement (per 1 score)	0.17 (0.06)	.006	0.16 (0.11)	.13	0.29 (0.13)	.03	0.28 (0.09)	.001	0.23 (0.09)	.012	10.2 (2.9)	.005	0.64 (0.36)	.072

Note

Adjusted for age, sex, household income, education, occupation, job stress, support of supervisor and colleague, K6, workplace and total energy intake.

Abbreviations: MUFA, monounsaturated fatty acids; PUFA, polyunsaturated fatty acids; SFA, saturated fatty acids.

Finally, we performed supplemental stratified analyses to examine whether the associations of work engagement with health behavior was modified by education level, occupational status, and household income (Table 3). These analyses were performed in order to test whether work engagement is beneficial mainly for high SES workers, or whether the health benefits are more generalized.

**TABLE 3:** The standardized regression coefficients (β) and scandalized error (*SE*) to estimate daily nutrition by work engagement by socioeconomic conditions

	Salt (g/d)		Lipid (g/d)						Dietary fiber (g/d)		Folate (μg/d)		Isoflavone (μg/d)	
	β	*SE*	SFA		MUFA		PUFA		β	*SE*	β	*SE*	Genistein	
			β	*SE*	β	*SE*	β	*SE*					β	*SE*
Years of education (years)^a^														
12 or less	0.26	0.07*	0.12	0.19	0.32	0.22	0.29	0.14	0.16	0.16	6.6	5.01	0.48	0.45
More than 13	−0.0002	0.11	0.41	0.11*	0.33	0.17	0.31	0.11*	0.23	0.12	11.6	3.7*	0.83	0.58
Occupation^b^														
Manager	0.15	0.13	0.60	0.26*	0.83	0.32*	0.63	0.2*	0.11	0.21	2.78	6.42	0.58	0.83
Non‐manual	0.24	0.09*	0.07	0.16	0.001	0.19	0.15	0.13	0.33	0.13*	12.5	4.01*	1.17	0.52*
Manual	0.03	0.14	0.29	0.24	0.75	0.3*	0.5	0.19*	0.23	0.22	15.5	7.0*	0.004	0.74
Others	0.12	0.23	−0.54	0.41	−0.11	0.47	0.02	0.31	−0.04	0.35	−0.45	11.5	−1.88	1.25
Household income (million JPY/year)^c^														
Less than 2.74	0.23	0.03*	0.25	0.34	0.37	0.4	0.2	0.25	0.41	0.25	17.1	8.87	0.17	0.97
2.75–5.49	0.28	0.19	0.29	0.14*	0.44	0.17*	0.37	0.11*	0.23	0.12	11.1	3.8*	0.77	0.45
More than 5.50	0.13	0.08	−0.15	0.22	0.04	0.28	0.22	0.18	0.12	0.18	5.1	5.45	0.67	0.73

Abbreviations: MUFA, monounsaturated fatty acids; PUFA, polyunsaturated fatty acids; SFA, saturated fatty acids.

^a^ Adjusted for age, sex, household income, occupation, job stress, support of supervisor and colleague, K6, workplace and total energy intake.

^b^ Adjusted for age, sex, household income, education, job stress, support of supervisor and colleague, K6, workplace and total energy intake.

^c^ Adjusted for age, sex, education, occupation, job stress, support of supervisor and colleague, K6, workplace and total energy intake.

* <.05.

A two‐tailed *p*‐value < .05 was considered statistically significant in the analyses. All analyses were performed using SPSS 21.0 computer software for Windows (IBM SPSS Japan Inc, Tokyo, Japan).

## 3 RESULTS

Characteristics of the participants are summarized in Table 1. The mean age was 43.4 ± 9.7 years, and 89.7% of the participants were men. The mean work engagement score was 2.9 ± 1.0. The average consumption of each nutrient was as follows: Salt: 10.4 ± 3.2 g/d; SFA: 12.7 ± 5.3 g/d; MUFA: 18.0 ± 6.9 g/d; PUFA: 12.8 ± 5.3 g/d; dietary fiber: 10.1 ± 4.2 g/d; Folate: 295.8 ± 126.9 μg/d; and Isoflavone: 16.5 ± 13.1 μg/d.

In the unadjusted model, there were significant associations between a higher work engagement score and higher salt intake, as well as higher intake of SFA, MUFA, PUMA, dietary fiber, folate, and polyphenol. In the adjusted model, higher work engagement was significantly positively associated with higher intake of salt (β = 0.17, *SE* = 0.06, *P* = .006), MUFA (β = 0.29, *SE* = 0.13, *P* = .03), PUMA (β = 0.28, *SE* = 0.09, *P* = .001), dietary fiber (β = 0.23, *SE* = 0.09, *P* = .012), and folate (β = 10.2, *SE* = 2.9, *P* = .005) consumption, but not SFA (β = 0.16, *SE* = 0.11, *P* = .13) or isoflavone (β = 0.64, *SE* = 0.36, *P* = .072).

The association between work engagement and daily consumption of each nutrient within the strata of high versus low socioeconomic status are shown in Table 3.

Among participants with more than 13 years of education, there was a significant association between higher work engagement and higher consumption of SFA, PUFA and folate. However, among participants with less than 12 years of education, there was an association between high work engagement and high salt intake.

We next stratified the analyses by occupational grade. Among managers, higher work engagement was correlated with higher intake of SFA, MUFA, and PUFA. Among non‐manual workers, work engagement was correlated with higher salt intake, as well as dietary fiber, folate, and isoflavone.

Lastly, we stratified the analyses by levels of household income. Among participants with low income (less than 2.74 million JPY), work engagement was correlated with higher salt intake. Among higher income participants, work engagement was associated with higher intake of SFA, MUFA, PUFA, and folate.

## 4 DISCUSSION

In this cross‐sectional study, we found that higher work engagement was associated with healthier diet patterns among Japanese workers. Work engagement was correlated with higher intake of MUFA, PUFA, dietary fiber, and folate. The only exception to this pattern was the (unexpected) correlation between work engagement and higher salt intake. Interestingly, when we stratified our analyses according to levels of socioeconomic status, the correlation between work engagement and salt intake was primarily observed among low SES workers.

The underlying mechanism relating to work engagement and healthy behaviors is not fully understood. However, previous studies investigating an association between positive psychological well‐being and healthy diet behaviors provide some clues.^7^, ^8^ A random‐effects meta‐analysis found that more optimistic individuals tended to engage in healthier diet behaviors compared with less optimistic individuals.^7^ Psychological well‐being may lead to better health behaviors via a number of processes. For example, people with higher psychological well‐being are more likely to persist at achieving life goals, including the pursuit of optimum health status.^34^, ^35^ Higher psychological well‐being is positively associated with adaptive coping strategies to reduce or manage stressors or negative emotions.^34^, ^35^ Thus, psychological well‐being may spur individuals to adopt better health behaviors through goal setting, self‐efficacy, motivation, and self‐regulation.^36^ Standard theories of health behavior (eg Theory of Planned Behavior) emphasize that the adoption and maintenance of health habits depends not just on knowledge and attitudes toward the behavior, but also on the individual's motivation to adopt the behavior (expressed in terms of goal setting, or the formation of an intention to perform a behavior). In turn, motivation is influenced by cognitive factors such as control beliefs, that is, how confident the individual feels about their self‐efficacy to perform the behavior. An employee with high work engagement is characterized by someone who is motivated at work and enjoys their work. In turn, that is, work engagement is postulated to have carry‐over benefits in terms of increased motivation to achieve life goals (goal setting), higher self‐efficacy, as well as stress coping (self‐regulation). Our results were consistent with these previous studies investigating the association between positive psychological well‐being and healthy diet behaviors.

SES is an important moderator of the relation between work engagement and dietary behavior. In our sample, the correlation between work engagement and healthy dietary patterns was more strongly observed among educated and high‐income workers. By contrast, our unexpected finding of a correlation between work engagement and higher salt intake was primarily found among low SES workers.

According to previous studies: (a) there is a socioeconomic disparity in salt intake in Japan, with low SES households consuming more salt than high SES households,^37^ and (b) the major foods in the Japanese diet that contribute to high salt intake are ramen noodles and Japanese‐style curry rice.^38^ These foods are quick to prepare, cheap, and energy‐dense, and therefore very popular during lunch breaks as well as “fast food on the go” after work.

Due to the cross‐sectional design of our study, we cannot exclude the possibility that causality runs in the opposite direction, suggesting that a healthy diet promotes work engagement. In fact, a growing number of studies suggest that plant‐based diets promote psychological wellbeing.^10^ This suggests that the relation between work engagement and dietary habits is likely to be bi‐directional. A healthy diet promotes better mood and higher work engagement, and conversely, greater work engagement promotes healthier lifestyles. Future prospective investigations are needed to investigate these pathways.

Besides the cross‐sectional design, we note some other limitations to our study. First, our findings cannot be generalized to other populations. Workers in Japan tend to report lower work engagement scores compared to other countries.^39^ The association between work engagement and healthy diet behaviors may vary in other social contexts. Second, we did not investigate the association between work engagement and other nutrients such as plant‐based proteins. Third, we did not have further information to explain why low SES individuals who are engaged in their work would consume more salt than high SES workers. Finally, we could not adjust for a full set of potential confounders, such as company size, industrial category, detailed work duties, corporate social responsibility, or organizational justice, all of which may affect work engagement.^40^, ^41^ These are important areas for examination in future work.

In conclusion, we found that higher work engagement was associated with healthy diet patterns among Japanese workers. Further studies are needed to assess whether changes in work engagement are associated with changes in diet patterns.

## INFORMED CONSENT

Written informed consent was obtained from all participants.

## REGISTRY AND THE REGISTRATION NUMBER OF THE STUDY/TRIAL

None.

## ANIMAL STUDIES

None.

## CONFLICT OF INTEREST

The authors have no conflicts of interest to disclose.

## ETHICAL APPROVAL

This study was conducted according to the guidelines laid down in the Declaration of Helsinki and all procedures involving research study participants were approved by the Graduate School of Medicine and Faculty of Medicine at the University of Tokyo (No. 2772), Kitasato University School of Medicine Hospital (B12‐103) and the University of Occupational and Environmental Health, Japan (10‐004).
